# Discriminatory ability of perioperative heart rate variability in predicting postoperative complications in major urologic surgery: a prospective cohort study

**DOI:** 10.1038/s41598-024-62930-2

**Published:** 2024-05-25

**Authors:** Talia Ryan, Andrew M. Walker, David Liepert

**Affiliations:** Department of Anesthesiology, Perioperative and Pain Medicine Cumming School of Medicine, University of Calgary, Foothills Medical Center, 1403 29th St., Calgary, N.W. T2N 2T9 Canada

**Keywords:** Cardiology, Medical research

## Abstract

We aimed to determine if continuous perioperative heart rate variability (HRV) monitoring could improve risk stratification compared to a short preoperative measurement in radical cystectomy patients. Electrocardiography (ECG) recordings were collected continuously preoperatively to discharge in 83 patients. Two, 5-min ECG signal segments (preoperative and at 24-h post ECG placement) were analyzed offline to extract HRV metrics. HRV metric discriminatory ability to identify patients with 30-day postoperative complications were analyzed using receiver operating characteristics curves. Sixty participants were included for analysis of which 27 (45%) developed a complication within 30 days postoperative. HRV was reduced in patients with complications. Postoperative standard deviation NN intervals and root mean square of successive differences had area under the curves (AUC) of 0.67 (95% CI 0.54 to 0.81) and 0.68 (95% CI 0.54 to 0.82), respectively. Significant discriminatory abilities were also reported for postoperative frequency metrics of absolute low frequency (LF) [AUC = 0.65 (95% CI 0.51 to 0.79)] and high frequency (HF) powers [AUC = 0.69 (95% CI 0.55 to 0.83)] and total power [AUC = 0.66 (95% CI 0.53 to 0.80)]. Postoperative acquired HRV metrics demonstrated improved discriminatory ability. Our findings suggest that longer-term perioperative HRV monitoring presents with superior ability to stratify complication risk.

## Introduction

Heart rate variability (HRV) involves the monitoring of beat-to-beat variations in heart rate, the time elapsed between RR intervals on an electrocardiogram. These variations are controlled by the autonomic nervous system (ANS), thus HRV provides a functional assessment of the sympathetic and parasympathetic divisions of the ANS. Dysfunction of the ANS is indicative of myocardial ischemia, organ dysfunction in sepsis, and perioperative morbidity and mortality^1–4^, and is demonstrated by increased or decreased HRV^5^.

Patients requiring radical cystectomy represent a vulnerable surgical population. Evidence suggests that 20–57% of radical cystectomy patients will develop postoperative complications within 30 days of surgery^6^. The most common complications are gastrointestinal, infectious, wound, and cardiac complications, with the highest mortality in cardiopulmonary events and sepsis^6,7^. Assessment of perioperative risk in these patients is critical to enable early recognition, management, and potential prevention of complications^8^.

HRV monitoring is a promising, non-invasive tool that may improve our ability to identify patients at risk of or experiencing perioperative complications^9^. Initial studies evaluating HRV in the perioperative period have demonstrated associations between altered HRV parameters and perioperative complications including myocardial ischemia, hypotension, infection, prolonged length of stay, and overall prediction of morbidity and mortality^3,10–17^. However, the heterogeneity of methods and outcomes in studies assessing these postoperative complications does not allow for consensus on the viability of using HRV metrics as a prediction tool^18–21^. Interestingly, a recent study by Ernst et al., demonstrated a simple methodology which showed an association between brief preoperative HRV monitoring and postoperative complications^13^.

The primary objective of this study was to determine if perioperative HRV monitoring could identify 30-day postoperative complications in radical cystectomy patients. Specifically, we sought to report changes in HRV metrics extracted from 5-min electrocardiography (ECG) recorded segments collected immediately preoperative and 24-h post ECG device placement between those with and without complications. We hypothesized that decreased HRV would be associated with patients who developed a complication within 30-days of their radical cystectomy. Our secondary objective was to identify specific HRV parameters that provided prognostic ability to identify patients at risk of postoperative complications providing further credence to the use of HRV as a perioperative risk stratification tool.

## Methods

### Study design

This report follows the "Strengthening the Reporting of Observational studies in Epidemiology", the STROBE Statement^22^. The study protocol was approved by the Health Research Ethics Board of Alberta Cancer Committee, Edmonton, Alberta, Canada (HREBA.CC-18-0592). The study was conducted in accordance with relevant guidelines and regulations. This prospective cohort study considered all patients ≥ 18 years of age having radical cystectomy surgery at the Rockyview General Hospital, Calgary, Alberta, Canada between January 2019 and June 2021. Consent to screen was obtained in the preoperative assessment clinic for all patients requiring radical cystectomy. Informed consent was subsequently obtained for all subjects that met inclusion criteria prior to their surgical procedure. Exclusion criteria were patients who had surgery within the past month, were hemodynamically unstable, had atrial fibrillation or had a pacemaker or implantable cardioverter device.

We targeted 60 participants for our pilot study. This corresponded to an initial recruitment time frame of approximately 6–8 months based on our site frequency of radical cystectomy surgical procedures. Krajewski et al.^7^ reported a complication rate of 30% for radical cystectomies during the perioperative period and up to 60% at 90 days postoperative. Using our primary outcome of non-specific, all-cause complication incidence at 30 days postoperative, we assumed a rate of 40%. As such, the recruitment of 6 participants would ensure that we captured at least one participant with a 30-day postoperative complication at a 95% confidence level. Extrapolating this to 60 participants, we assumed that we would capture 10 participants with a 30-day postoperative complication. To account for potential ECG signal collection errors and loss to follow-up, we increased the number of recruited participants up to a maximum of 90.

## Procedure

All participants had a modified Biotricity Bioflux 1 (Biotricity, Redwood City, California, USA), a continuous, ambulatory cloud-based and Food and Drug Administration (FDA) approved ECG monitor applied preoperatively. Three wet contact lead ECG patches were applied at the left and right infraclavicular fossa and the lower chest to the left of the umbilicus. The monitor remained in place for the duration of hospital stay with intermittent charging performed daily. The device was connected and data was continuously collected at 1000 Hz from the time of ECG patch placement until discharge or participant request to remove. Consented participants were placed on the Enhanced Recovery After Surgery (ERAS) protocol for preoperative, intraoperative, and postoperative management^23^. Patients received balanced anesthesia including standard induction agents of propofol, opioid, neuromuscular blocker, maintenance with volatile anesthesia or hypnotic infusion, multimodal analgesia, and antiemetics. Patients were admitted to the urology unit for postoperative care.

ECG signals were analyzed offline to extract HRV metrics (see Supplemental Information S1) in both time [standard deviation of normal NN intervals (SDNN), root mean squared standard deviation (RMSSD)] and frequency [absolute very low frequency (VLF) power, absolute low frequency (LF) power, LF power in normalized units, absolute high frequency (HF) power, HF power in normalized units and total power] domains using Kubios HRV Premium software version 3.4.1 (Kubios Oy, Kuopio, Finland) and in accordance with The Task Force of European Society of Cardiology and the North American Society of Pacing and Electrophysiology standards with respect to HRV analysis^24^.

We targeted two time points to acquire a 5-min ECG segment. We attempted to standardize environmental conditions during acquisition. Our first 5-min segment was targeted preoperatively between 10 min prior to up to the time of anesthesia induction. At this time, all participants were prepped for surgery and lying supine in the operating suite. Our second 5-min segment target was 24-h ± 1 h after ECG device placement. Within each target window, the ECG signal was manually visualized to locate the 5-min segment with the lowest percentage of signal artifact. R-R interval detrending was completed using a smoothness priors regularisation with a cut-off frequency of 0.035 Hz. Artifacts were corrected using Kubios automatic artifact correction algorithm for beat correction^25^. Processing of HRV metrics from participant ECG signals was restricted to those presenting with < 10% artifact^26^.

### Data collection

Clinical data was collected at admission and during daily follow-up using a clinical report form and electronic medical records. Demographics, past medical history, medications, and complications up to 30 days postoperative were recorded. Acquired ECG data was uploaded for offline analysis at time of discharge.

Postoperative complications including infection (pneumonia, urinary tract infection, surgical site infection), stroke, ileus, myocardial infarction, deep vein thrombosis (including pulmonary embolism) and death were identified at time of discharge and reviewed at 30 days postoperative using electronic medical records, discharge summary letters, and re-admission documentation. These specific complications were tracked due to their common presentation in this surgical population^6^. Complications were defined in accordance with nationally established standards (see Supplemental Information S2).

### Analyses

Continuous participant characteristics were tested for normality using the Shapiro–Wilk test and presented using mean ± standard deviation. Binary characteristics were presented using frequency (percentage). Comparisons between groups were completed using independent samples t-tests, chi-square tests for associations or Fisher’s exact tests as appropriate. Differences in pre- and postoperative HRV metrics between participants with and without complications were explored using Mann–Whitney U tests and presented as median[interquartile range(min–max)]. The discriminatory power of pre- and postoperative HRV metrics to predict a 30-day postoperative complication was plotted using non-parametric receiver operator characteristic (ROC) curves with accompanying area under the curve (AUC) values and 95% confidence intervals (CI) generated using 2000 bootstrapped replicates. Sensitivity, specificity, positive (PPV) and negative predictive values (NPV) with 95% CI were reported corresponding to cut-off values that maximized the sum of specificity and sensitivity (Youden Index). *p* < 0.05 was considered significant. Demographic and group comparison statistical analyses was completed using SPSS version 25.0 software (IBM, Armonk, NY, USA). ROC curve analysis and the calculation of sensitivity, specificity, PPV, and NPV values at the Youden Index for each metrics were completed using R Studio version 2023.03.0 with R statistical software version 4.3.0 (R Foundation for Statistical Computing, Vienna, Austria) using pROC (version 1.18.5) and epiR (version 2.0.74) packages^27,28^.

## Results

**Figure 1:**
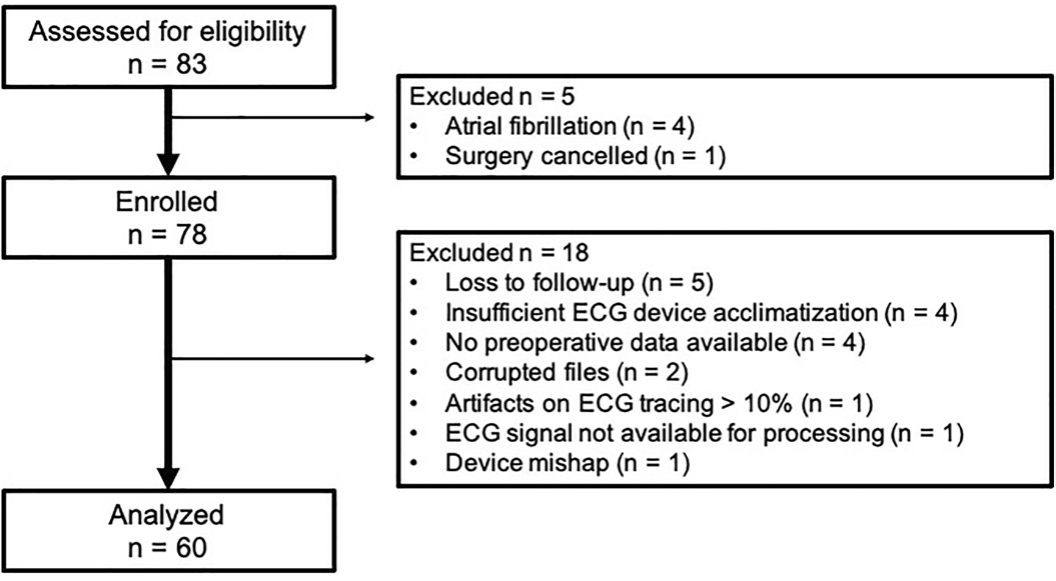
Study flow chart.

Sixty participants of 83 assessed for eligibility were included for analysis of which 27 (45%) developed a complication within 30 days postoperative (Fig. 1, Table 1). Complications included 11 surgical site infections, nine urinary tract infections, two cases of pneumonia, and one each of myocardial infarction, supraventricular tachycardia, stroke/transient ischemic attack, pulmonary embolism, and seizure. No significant differences were noted in patient characteristics between groups except for increased body mass index (BMI) in those with complications (Table 1). No significant differences were found in preoperative HRV metrics between participants who did and did not develop a 30-day postoperative complication (Table 2). Median postoperative SDNN (ms) values were 19.7[9.7–34.7(5.6–87.4)] and 12.5[7.5–19.0(3.8–38.2)] in participants without and with complications, respectively (*p* = 0.022) (Table 2). Median postoperative RMSSD (ms) values were 17.0[9.3–27.3(4.9–78.8)] and 8.7[6.1–14.7(4.2–68.5)] in those without and with complications (*p* = 0.020) (Table 2). Postoperative absolute LF (*p* = 0.043) and HF power (*p* = 0.012), and total power (*p* = 0.029) were significantly lower in participants that developed complications (Table 2).

**Table 1:** Participant characteristics of those developing and not developing a complication 30-days postoperative from radical cystectomy surgery.

	All participants (n = 60)	No Complications (n = 33)	Complications (n = 27)	*p* value^a^
Age (years)	69.5 ± 7.9	69.8 ± 7.3	69.1 ± 8.7	0.719
Gender (male)	43 (71.7)	27 (81.8)	16 (59.3)	0.054
Height (cm)	170.1 ± 9.0	171.4 ± 8.6	168.4 ± 9.4	0.202
Weight (kg)	82.7 ± 16.9	79.6 ± 14.6	86.4 ± 18.9	0.124
Body mass index (BMI) (kg/m^2^)	28.5 ± 5.1	27.1 ± 4.3	30.3 ± 5.5	0.014
Comorbidity				
Hypertension	39 (65.0)	19 (57.6)	20 (74.1)	0.183
Coronary artery disease	7 (11.7)	5 (15.2)	2 (7.4)	0.442
Diabetes mellitus type I	1 (1.7)	0 (0.0)	1 (3.7)	0.450
Diabetes mellitus type II	12 (20.0)	5 (15.2)	7 (25.9)	0.299
Chronic obstructive pulmonary disorder	10 (16.7)	5 (15.2)	5 (18.5)	0.742
Atrial fibrillation	0 (0.0)	0 (0.0)	0 (0.0)	n/a
Obesity (BMI > 30)	24 (40.0)	10 (30.3)	14 (51.9)	0.090
Medications				
β-blocker	9 (15.0)	5 (15.2)	4 (14.8)	1.000
ACE-inhibitor	13 (21.7)	6 (18.2)	7 (25.9)	0.469
Calcium channel blocker	10 (16.7)	4 (12.1)	6 (22.2)	0.322
Angiotensin II receptor blocker	20 (33.3)	10 (30.3)	10 (37.0)	0.582
Digitalis	1 (1.7)	1 (3.0)	0 (0.0)	1.000
Nitrate	1 (1.7)	1 (3.0)	0 (0.0)	1.000
Diuretics	7 (11.7)	5 (15.2)	2 (7.4)	0.442
Statins	29 (48.3)	17 (51.5)	12 (44.4)	0.586

Data presented as mean ± standard deviation or frequency (percentage).

*n* number, *ACE* angiotensin-converting enzyme.

^a^*p* values in relation to comparisons between patients with no complications or complications at 30-days postoperative.

**Table 2:** Heart rate variability (HRV) metrics collected prior to anesthesia induction and 24-h post-ECG monitor placement in participants who did and did not develop complications within 30 days postoperative.

	Prior to anesthesia induction		
	No complication	Complication	*p* value
SDNN (ms)	27.0 [15.8–53.0 (4.8–182)]	18.4 [12.7–26.6 (6.1–85.9)]	0.056
RMSSD (ms)	19.9 [12.9–42.4 (4.4–237)]	16.5 [9.2–26.3 (5.5–68.8)]	0.128
Absolute VLF power (ms^2^)	57.1 [20.1–121 (1.2–551)]	28.2 [11.9–69.4 (0.4–1703)]	0.095
Absolute LF power (ms^2^)	209 [92.3–523 (15.3–11,678)]	219 [52.6–436 (7.0–7210)]	0.705
Normalized LF (nu)	71.7 [56.2–82.0 (26.8–93.4)]	71.2 [65.4–80.3 (3.3–96.4)]	0.864
Absolute HF power (ms^2^)	112 [26.9–316 (4.9–10,104)]	96.6 [26.8–228 (3.0–955)]	0.518
Normalized HF (nu)	28.3 [18.0–43.8 (6.3–72.7)]	28.8 [19.7–34.5 (3.6–96.2)]	0.841
Total Power (ms^2^)	377 [141–864 (29.5–21,956)]	316 [122–656 (30.9–9868)]	0.508
24-h post-ECG monitor placement			
SDNN (ms)	19.7 [9.7–34.7 (5.6–87.4)]	12.5 [7.5–19.0 (3.8–38.2)]	0.022
RMSSD (ms)	17.0 [9.3–27.3 (4.9–78.8)]	8.7 [6.1–14.7 (4.2–68.5)]	0.020
Absolute VLF power (ms^2^)	29.6 [9.8–119 (0.6–701)]	19.4 [4.8–50.6 (0.6–152)]	0.120
Absolute LF power (ms^2^)	170 [34.7–402 (3.8–6922)]	56.0 [26.0–217 (4.3–823)]	0.043
Normalized LF (nu)	66.3 [51.3–78.5 (19.5–90.3)]	78.0 [59.4–82.7 (22.6–91.7)]	0.152
Absolute HF power (ms^2^)	85.4 [20.9–296 (4.2–2071)]	25.9 [8.0–79.0 (2.0–868)]	0.012
Normalized HF (nu)	33.7 [21.5–48.6 (9.7–80.5)]	22.0 [17.2–40.6 (8.2–77.4)]	0.152
Total Power (ms^2^)	370 [83.3–778 (8.6–9243)]	107 [49.5–373 (10.7–1788)]	0.029

Data presented as median [interquartile range (min–max)].

*SDNN* standard deviation of NN intervals, *RMSSD* root mean squared standard deviation, *VLF* very low frequency, *LF* low frequency, *HF* high frequency, *ms* milliseconds, *nu* normalized units, *ECG* electrocardiogram.

**Figure 2:**
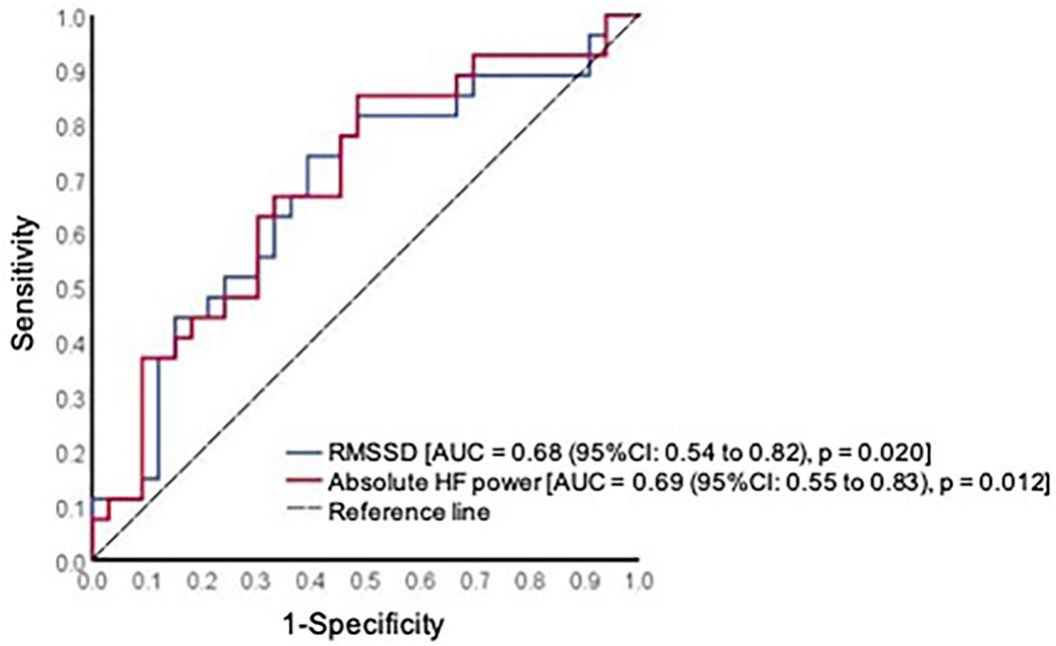
Non-parametric receiver operating characteristic (ROC) curves displaying the discriminatory power of postoperative root mean squared standard error (RMSSD) and postoperative absolute high frequency (HF) power in predicting 30-day postoperative complications. AUC, area under the curve.

The discriminatory power of HRV metrics is presented in Table 3 and Fig. 2. Except absolute VLF, all HRV metrics acquired postoperative presented with superior discriminatory ability compared to preoperative measures (Table 3). Postoperative SDNN and RMSSD (Fig. 2) presented with comparable AUCs of 0.67 (95% CI 0.54 to 0.81) and 0.68 (95% CI 0.54 to 0.82), respectively (Table 3). Significant predictive abilities were also found for postoperative absolute LF [AUC = 0.65 (95% CI 0.51 to 0.79)] and HF power [AUC = 0.69 (95% CI 0.55 to 0.83)] (Fig. 2), and total power [AUC = 0.66 (95% CI 0.53 to 0.80)] (Table 3). Given improved discriminatory abilities, reported sensitivity, specificity, PPV and NPV using clinical cut-off values were restricted to postoperative measurements (Table 4).

**Table 3:** Discriminatory ability of heart rate variability (HRV) metrics acquired prior to anesthesia induction and 24-h post-ECG monitor placement for participants who did and did not develop complications within 30 days postoperative.

	Prior to anesthesia induction		24-h post ECG monitor placement	
	AUC (95% CI)	*p* value	AUC (95% CI)	*p* value
SDNN (ms)	0.64 (0.50 to 0.79)	0.056	0.67 (0.54 to 0.81)	0.022
RMSSD (ms)	0.62 (0.47 to 0.76)	0.128	0.68 (0.54 to 0.82)	0.020
Absolute VLF power (ms^2^)	0.63 (0.48 to 0.77)	0.095	0.62 (0.48 to 0.76)	0.120
Absolute LF power (ms^2^)	0.53 (0.38 to 0.68)	0.705	0.65 (0.51 to 0.79)	0.043
Normalized LF (nu)	0.51 (0.36 to 0.66)	0.864	0.61 (0.46 to 0.75)	0.152
Absolute HF power (ms^2^)	0.55 (0.40 to 0.70)	0.518	0.69 (0.55 to 0.83)	0.012
Normalized HF (nu)	0.52 (0.37 to 0.67)	0.841	0.61 (0.46 to 0.75)	0.152
Total Power (ms^2^)	0.55 (0.40 to 0.70)	0.508	0.66 (0.53 to 0.80)	0.029

*ECG* electrocardiogram, *AUC* area under the curve, *CI* confidence interval, *SDNN* standard deviation of NN intervals, *RMSSD* root mean squared standard deviation, *VLF* very low frequency, *LF* low frequency, *HF* high frequency, *ms* milliseconds, *nu* normalized units.

**Table 4:** Postoperative heart rate variability (HRV) metric cut-off values for the prediction of a complication within 30 days postoperative. Presented cut-off values correspond to the maximum value of the sum of the sensitivity and specificity (Youden Index) for each respective time and frequency domain HRV receiver operator characteristic (ROC) curve.

	Cut-off Value	Sensitivity (95% CI)	Specificity (95% CI)	PPV (95% CI)	NPV (95% CI)
SDNN (ms)	≤ 19.3	81 (63 to 92)	55 (38 to 70)	59 (43 to 74)	78 (58 to 90)
RMSSD (ms)	≤ 14.3	74 (55 to 87)	61 (44 to 75)	61 (44 to 75)	74 (55 to 87)
Absolute VLF power (ms^2^)	≤ 52.1	78 (59 to 89)	45 (30 to 62)	54 (39 to 68)	71 (50 to 86)
Absolute LF power (ms^2^)	≤ 136.3	74 (55 to 87)	61 (44 to 75)	61 (44 to 75)	74 (55 to 87)
Normalized LF (nu)	≥ 77.6	56 (37 to 72)	73 (56 to 85)	62 (43 to 79)	67 (50 to 80)
Absolute HF power (ms^2^)	≤ 85.2	85 (68 to 94)	52 (35 to 67)	59 (43 to 73)	81 (60 to 92)
Normalized HF (nu)	≤ 22.4	56 (37 to 72)	73 (56 to 85)	62 (43 to 79)	67 (50 to 80)
Total Power (ms^2^)	≤ 296.3	74 (55 to 87)	61 (44 to 75)	61 (44 to 5)	74 (55 to 87)

*CI* confidence interval, *PPV* positive predictive value, *NPV* negative predictive value, *SDNN* standard deviation of NN intervals, *RMSSD* root mean squared standard deviation, *VLF* very low frequency, *LF* low frequency, *HF* high frequency, *ms* milliseconds, *nu* normalized units.

## Discussion

In this prospective cohort study on 30-day postoperative complications in radical cystectomy patients, perioperative HRV was analyzed in the pre- and postoperative periods. We found significant differences in postoperative HRV metrics between participants who did and did not develop postoperative complications. We found no difference in preoperative HRV metrics between those who did and did not develop complications. This contrasts with Ernst et al.^13^ who reported significant preoperative differences in RMSSD and total power in those who developed complications after hip fracture surgery. However, our cohort included elective surgery patients as opposed to patients requiring urgent surgical repair^13^. Our finding of no preoperative differences may be explained by the timing of physiological stressors on the patient. Those presenting for urgent hip fracture surgery likely had a high degree of preoperative physiological stress. Our cohort of elective surgery patients likely presented with greater physiological stress after their highly invasive radical cystectomy procedure.

Postoperative SDNN, RMSSD, absolute LF and HF power and total power were significantly lower in patients who developed complications. Lower SDNN has been associated with increased morbidity and mortality in various clinical populations^4,29–31^. Reduced RMSSD has been associated with complications in the post-surgical population and may be indicative of lower parasympathetic activity^13^. A study by Cha et al. reported that SDNN, RMSSD, LF and HF power and total power were significant in predicting adverse cardiovascular outcomes in diabetic patients^32^. HRV has been associated with frailty, age-related impairment of hemostatic mechanisms, resulting in critical loss of physiologic response to stressors^33^. This suggests that changes in HRV are associated with the ANS and represents alterations in general physiologic performance.

Our findings showed improved discriminatory power of postoperative HRV metrics including SDNN, RMSSD, absolute LF, absolute HF, and total power compared to preoperative values. The use of cut-off values with associated sensitivity and specificity may allow for simplified clinical utility of HRV metrics in practice through the use of real-time monitoring. As such, HRV could be a useful indicator to identify patients at risk of postsurgical complications through risk stratification via monitoring of HRV metrics and their relationship with established cut-off values. Though vital signs are essential in the monitoring and management of patients, the application of HRV as a measure of sympathetic and parasympathetic nerve system balance may demonstrate increased utility. Perioperative HRV monitoring assessed in real-time may enhance detection and early management of patients at risk of developing postoperative complications. Kasaoka et al. demonstrated such using real- time HRV metrics (LF, HF and LF/HF ratio) in the intensive care unit to immediately assess and investigate clinical conditions of critically ill patients^34^.

Our study is not without limitations. Complications may be underreported if they did not occur in hospital or community office without access to the Alberta province wide electronic medical data system. Our study used 5-min HRV segments for short term analysis^35^. Though an attractive approach for data analysis and clinical application, this approach is comparatively more sensitive to artifact than using longer ECG segments. We standardized our preoperative 5-min ECG segment between 10 min prior to up to the time of anesthesia induction when patients were supine. No such explicit standardization occurred for our 24-h post-ECG monitor placement measurements. As such, there is the possibility that patients could have been ambulatory given ERAS recommendations of early mobilization (up to 2-h postoperative day 0)^23^.

Our study was not powered to detect a specific difference between preoperative and postoperative HRV metrics, between participants who did and did not develop complications or on the discriminatory ability of HRV metrics. As such, we cannot conclude with certainty which HRV metric has the strongest discriminatory ability for risk stratification purposes. Our results suggest that postoperative monitoring of HRV provides improved risk stratification capabilities compared to a single short preoperative recording. However, our findings are not necessarily translatable to other surgical populations that have lower risks of postoperative complications or surgeries that may be associated with high preoperative physiological stress. We cannot discount the possible influence of both collected and unobserved confounding factors on reported differences in HRV metrics between groups and their discriminatory ability. Indeed, several participants were associated with comorbidities and medications that can affect the ANS and HRV. Except for BMI, no statistically significant differences were noted between groups. Multivariable logistic regression can be used to address confounding factors. However, following the often used 10 events per variable criteria for multivariable regression, where in this instance a complication represents an event, we are restricted to a maximum of two variables in any such model^36^.

Although these limitations must be considered when synthesizing our results, this work has developed a foundation for larger appropriately powered studies to determine if long-term monitoring is indeed superior to preoperative recordings across a variety of surgical and perioperative settings. Our analysis of HRV metric discriminatory abilities has allowed for the initial presentation of clinical cut-offs values to predict who will and will not develop postoperative complications. At this point, these should not be considered established reference values for risk stratification. However, further rigorous exploration of such values and implementation into clinical monitoring is strongly recommended to improve interpretation of HRV metrics (is patient above or below cut-off value?) while providing real-time visualization of current clinical status and potential future destabilization.

## Conclusion

HRV was reduced in participants who developed complications 30-days postoperative. Postoperative HRV metrics showed improved discriminatory ability of participants who did and did not develop postoperative complications within 30 days. This suggests that longer-term perioperative HRV monitoring has the improved ability to stratify complication risk with respect to our specific cohort. Specific HRV metrics including postoperative SDNN, RMSSD, absolute LF and HF power, and total power demonstrated significant discriminatory power for development of complications. Our analysis allowed for the presentation of preliminary clinical cut-off values that presents an attractive tool for future risk stratification. The presented values should not be considered established reference values, but used a guide to further develop the concept of HRV as a real-time clinical tool for clinical status monitoring. The analysis and interpretation of HRV metrics can be difficult for the clinician to process. However, future appropriately powered studies is a worthy endeavour that would improve interpretation and permit real-time clinical assessment and risk stratification.

### Supplementary Information

Supplementary Information.

## Data Availability

The data that support the findings of this study are not available without the prior written consent of Alberta Health Services. Data are however available from the authors upon reasonable request and with the permission of Alberta Health Services. Please contact the corresponding author: Dr. Talia Ryan, Department of Anesthesiology, Perioperative and Pain Medicine, Cumming School of Medicine, University of Calgary, Foothills Medical Centre, 1403 29^th^ St. N.W., T2N 2T9, CANADA.

## Abbreviations

HRV: Heart rate variability
ANS: Autonomic nervous system
ECG: Electrocardiography
FDA: Food and Drug Administration
ERAS: Enhanced Recovery After Surgery
SDNN: Standard deviation of normal NN intervals
RMSSD: Root mean squared standard deviation
VLF: Very low frequency
LF: Low frequency
HF: High frequency
ROC: Receiver operator characteristic
AUC: Area under the curve
CI: Confidence interval
PPV: Positive predictive value
NPV: Negative predictive value
BMI: Body mass index
