# Supplementary material for: Discriminatory ability of perioperative heart rate variability in predicting postoperative complications in major urologic surgery: a prospective cohort study
Source: Sci Rep. 2024 May 25;14:11965. doi: 10.1038/s41598-024-62930-2 (PMC11127941; doi:10.1038/s41598-024-62930-2)
Supplement: Supplementary file 1 — Supplementary Information. [file 41598_2024_62930_MOESM1_ESM.docx]

**Supplemental Information**

S1. Definitions of reported heart rate variability (HRV) metrics

| ^a^**HRV metric reported** | **Definition**^1,2^ |
| --- | --- |
| SDNN | - Standard deviation of interbeat intervals after abnormal beat removal (ms) - Influenced by both the sympathetic and parasympathetic nervous systems |
| RMSSD | - Root mean square of interbeat intervals between all successive heartbeats (ms) - Values are more highly influenced by the parasympathetic nervous system than SDNN |
| Absolute VLF power | - Absolute power (ms^2^) of the very low frequency band enveloping frequencies of 0.0033-0.04 Hz (periods between 25 and 300 seconds) - Associated with sympatho-vagal balance |
| Absolute LF power | - Absolute power (ms^2^) of the low frequency band encompassing frequencies of 0.04-0.15 Hz (periods between 7 and 25 seconds) - May be associated with parasympathetic and sympathetic nervous systems, baroreflex activity or primarily by parasympathetic nervous system |
| Normalized LF | - Presentation of low frequency power in normalized units by dividing absolute low frequency power by the sum of absolute low and high frequency power |
| Absolute HF power | - Absolute power (ms^2^) of the high frequency band enveloping frequencies of 0.15-0.40 Hz (periods between 25 and 300 seconds) - Representative of parasympathetic activity |
| Normalized HF | - Presentation of high frequency power in normalized units by dividing absolute high frequency power by the sum of absolute low and high frequency power |
| Total Power | - Summed very low, low and high frequency absolute powers (ms^2^) |

^a^For normal values of HRV in healthy adults using short-term measurements, please see summarized values provided by Shaffer and Ginsberg (1, Table 6) and referenced studies within.

1. Shaffer F, Ginsberg JP. An overview of heart rate variability metrics and norms. Front Public Health. 2017; **5**: doi:10.3389/fpubh.2017.00258.
2. Usui H, Nishida Y. The very low-frequency band of heart rate variability represents the slow recovery component after a mental stress task. PloS One. 2017; **12**: e0182611.

S2. Diagnoses criteria for postoperative complications

- **Pneumonia** was diagnosed in accordance with nationally established standards including fever, leukocytes, new onset purulent sputum and isolated pathological specimen, chest radiograph with new or progressive infiltrates, consolidation, or effusion. (1)
- **Urinary tract infection** was based on clinical diagnosis and associated clinical symptoms of fever, dysuria, and bacteriuria. Surgical site infections were diagnosed based on the American College of Surgeons National Surgical Quality Improvement Program (ACS-NSQIP) definition, “an infection that occurs within 30 d after the operation and requires at least one of the following: purulent drainage from the superficial incision; organisms isolated from an aseptically obtained culture of fluid or tissue from the incision; at least one of the following signs or symptoms of infection: pain or tenderness, localized swelling, redness, or heat, or fever (>8°C).” (2)
- **Stroke** was diagnosed according to national and international guidelines. (3-4)
- **Myocardial infarction** was diagnosed following consultation with internal medicine or cardiology according to the Fourth Universal Definition of myocardial infarction diagnostic criteria: new onset of symptoms of myocardial ischemia and elevated cardiac troponin values in addition to one of the following new significant ECG changes, development of pathologic Q waves, imaging evidence of new loss of viable myocardium or new regional wall motion abnormalities, or the identification of thrombus by angiography. (5)
- **Deep vein thrombosis and pulmonary embolism** were diagnosed based on clinical presentation and diagnostic imaging including limb ultrasonography and computed tomography pulmonary angiography.

1. Rotstein C, Evans G, Born A, et al. Clinical practice guidelines for hospital-acquired pneumonia and ventilator-associated pneumonia in adults. Canadian Journal of Infectious Diseases and Medical Microbiology. 2008; **19**: 19-52.
2. Ortega G, Rhee DS, Papandria DJ, et al. An evaluation of surgical site infections by wound classification systems using the ACS-NSQIP. Journal of Surgical Research. 2012; **174**: 33-38.
3. Boulanger JM, Lindsay MP, Gubitz G, et al. Canadian stroke best practice recommendations for acute stroke management: prehospital, emergency department, and acute impatient stroke care, update 2018. International Journal of Stroke. 2018; **13**: 949-984.
4. Powers WJ, Rabinstein AA, Ackerson T, et al. Guidelines for the early management of patients with acute ischemic stroke: 2019 update to the 2018 guidelines for the early management of acute ischemic stroke: a guideline for healthcare professionals from the American Heart Association/American Stroke Association. Stroke. 2019; **50**: e344-418.
5. Thygesen K, Alpert JS, Jaffe AS, et al. on behalf of the Joint European Society of Cardiology (ESC)/American College of Cardiology (ACC)/American Heart Association (AHA)/World Heart Federation (WHF) Task Force for the Universal Definition of Myocardial Infarction. Fourth universal definition of myocardial infarction. Journal of the American College of Cardiology. 2018; **72**: 2231-2264.
